# miR-93 functions as an oncomiR for the downregulation of PDCD4 in gastric carcinoma

**DOI:** 10.1038/srep23772

**Published:** 2016-03-29

**Authors:** Hongwei Liang, Feng Wang, Danping Chu, Weijie Zhang, Zhicong Liao, Zheng Fu, Xin Yan, Hao Zhu, Wen Guo, Yujing Zhang, Wenxian Guan, Xi Chen

**Affiliations:** 1 State Key Laboratory of Pharmaceutical Biotechnology, NJU Advanced Institute for Life Sciences, Jiangsu Engineering Research Center for MicroRNA Biology and Biotechnology, School of Life Science, Nanjing University, Nanjing, Jiangsu 210093, China; 2 Department of General Surgery, The Affiliated Drum Tower Hospital of Medical School of Nanjing University and Nanjing Multi-center Biobank, Nanjing, Jiangsu 210008, China; 3 Department of Cardio-Thoracic Surgery, The Affiliated Drum Tower Hospital of Medical School of Nanjing University and Nanjing Multi-center Biobank, Nanjing, Jiangsu 210008, China; 4 Department of Respiratory Medicine, The Affiliated Drum Tower Hospital of Medical School of Nanjing University and Nanjing Multi-center Biobank, Nanjing, Jiangsu 210008, China; 5 Department of Gastroenterology, The Affiliated Drum Tower Hospital of Medical School of Nanjing University and Nanjing Multi-center Biobank, Nanjing, Jiangsu 210008, China; 6 Department of Endocrinology, Nanjing Municipal Hospital for Governmental Organizations, Nanjing, Jiangsu 210018, China

## Abstract

Programmed cell death 4 (PDCD4), as a tumor suppressor gene, is frequently reduced in a variety of tumors, including gastric cancer. Previous findings have indicated that PDCD4 participates in tumorigenesis through the regulation of apoptosis, but the molecular basis of this process has not been fully elucidated, and no studies have shown the upstream regulation of this gene in gastric cancer. In this study, we used bioinformatics analysis to search for miRNAs that could potentially target PDCD4 and identified miR-93 as a candidate. Moreover, we observed the inverse correlation between miR-93 and PDCD4 protein levels, but not mRNA levels, in human gastric cancer tissues. We further experimentally validated PDCD4 as the direct target of miR-93 by evaluating PDCD4 expression in gastric cancer cells after the overexpression or knockdown of miR-93. Additionally, the biological consequences of targeting PDCD4 through miR-93 were examined using cell apoptosis assays *in vitro*. We demonstrated that the repression of PDCD4 through miR-93 suppressed the apoptosis of gastric cancer cells. Finally, we revealed that miR-93 promoted the development of gastric tumor growth in xenograft mice by negatively regulating PDCD4. Taken together, the findings of the present study indicated the oncogenic role of miR-93 in gastric cancer tumorigenesis through targeting PDCD4, particularly in apoptosis.

Gastric cancer is one of the most common human cancers. Although the incidence and mortality of this disease have decreased worldwide over the past 20 years, gastric cancer remains the fourth most common and the second most lethal cancer worldwide^1^. Knowledge of the molecular mechanisms underlying gastric cancer has major importance and might provide novel strategies to improve the survival and quality of life of gastric cancer patients. Therefore, it is important to understand the molecular basis of gastric cancer and explore new therapeutic agents.

With substantial advances in our understanding of tumor biology, key oncogenes and tumor suppressor genes involved in mediating cancer growth and progression have become clear. These genes offer new targets for biological therapies. One such target is PDCD4 (programmed cell death 4). PDCD4 expression is commonly reduced in a variety of tumors^2^,^3^,^4^,^5^. As a tumor suppressor gene, PDCD4 is upregulated after the initiation of apoptosis^6^,^7^. In gastric cancer, PDCD4 regulates apoptosis through the downregulation of FLICE-inhibiting protein (FLIP), a negative regulator of apoptosis^5^. However, to the best of our knowledge, no studies have shown the upstream regulation of this gene in gastric cancer.

MicroRNAs (miRNAs) are small non-coding RNAs of 20 ~ 22 nucleotides. These molecules repress gene expression through interactions with the 3′-untranslated regions (3′-UTRs) of mRNAs. miRNAs target more than 50% of all human protein-coding genes, playing numerous regulatory roles in many physiological and developmental processes, including development, differentiation, apoptosis and proliferation^8^. miR-93 is one of the miRNAs often seen upregulated in cancers^9^,^10^,^11^,^12^. Several studies have reported the upregulation of miR-93 in gastric cancer^13^,^14^, showing that miR-93 functions as an oncomiR to enhance cancer cell apoptosis^10^,^15^,^16^,^17^,^18^. However, the molecular mechanisms underlying the contribution of miR-93 to the development and progression of gastric cancer remain elusive.

In the present study, we identified PDCD4 as a direct target of miR-93. The potential role of miR-93 as an oncomiR of gastric cancer through PDCD4 targeting in apoptosis has been experimentally validated.

## Materials and Methods

### Cells and human tissues

The human gastric cancer cell lines AGS were purchased from the Shanghai Institute of Cell Biology, Chinese Academy of Sciences (Shanghai, China). AGS cells were cultured in RPMI 1640 supplemented with 10% fetal bovine serum (GIBCO, CA, USA) and incubated in 5% CO_2_ at 37 °C in a water-saturated atmosphere. The gastric cancer and paired normal adjacent tissues were derived from patients undergoing a surgical procedure at the Affiliated Drum Tower Hospital of Nanjing University (Nanjing, China). All protocols concerning the use of patient samples in this study were approved by the Medical Ethics Committee of the Affiliated Drum Tower Hospital of Nanjing University (Nanjing, China). A signed consent form was obtained from each donor. The tissue fragments were immediately frozen in liquid nitrogen at the time of surgery and stored at −80 °C. The clinical features of the patients are listed in Supplementary Table 1. Study protocol was approved by the Medical Ethics Committee of the Affiliated Drum Tower Hospital of Nanjing University (Nanjing, China) and all experiments were performed in accordance with approved guidelines of the Affiliated Drum Tower Hospital of Nanjing University (Nanjing, China).

### RNA isolation and quantitative RT-PCR

Total RNA was extracted from the cultured cells and human tissues using TRIzol reagent (Invitrogen, Carlsbad, CA) according to the manufacturer’s instructions. Assays to quantify miRNAs were performed using TaqMan miRNA probes (Applied Biosystems, Foster City, CA) according to the manufacturer’s instructions. Briefly, 1 μg of total RNA was reverse-transcribed to cDNA using AMV reverse transcriptase (TaKaRa, Dalian, China) and a stem-loop RT primer (Applied Biosystems). The following reaction conditions were used: 16 °C for 30 min, 42 °C for 30 min, and 85 °C for 5 min. Real-time PCR was performed using a TaqMan PCR kit on an Applied Biosystems 7300 Sequence Detection System (Applied Biosystems). The reactions were incubated in a 96-well optical plate at 95 °C for 10 min, followed by 40 cycles of 95 °C for 15 sec and 60 °C for 1 min. All reactions were run in triplicate. After the reaction, the cycle threshold (C_T_) data were determined using fixed threshold settings, and the mean C_T_ of the triplicate PCRs was determined. A comparative C_T_ method was used to compare each condition with the controls. The relative levels of the miRNAs in cells and tissues were normalized to U6. The amount of miRNA relative to the internal control U6 was calculated using the 2^−ΔΔCT^ equation, in which ΔΔC_T _= (C_T miRNA _− C_T U6_)_target_ − (C_T miRNA _− C_T U6_)_control_.

To quantify PDCD4 mRNA, 1 μg of total RNA was reverse-transcribed to cDNA using oligo dT and AMV reverse transcriptase (TaKaRa), performed using the following conditions: 42 °C for 60 min and 70 °C for 10 min. Subsequently, real-time PCR was performed using the RT product, SYBRGreen Dye (Invitrogen), and specific primers for PDCD4 and GAPDH. The sequences of the primers were PDCD4 (sense): 5′- TATGATGTGGAGGAGGTGGATGTGA-3′; PDCD4 (antisense): 5′- CCTTTCATCCAAAGGAAAAACTACAC-3′; GAPDH (sense): 5′-GATATTGTTGACATCAATGAC-3′; and GAPDH (antisense): 5′-TTGATTTTGGAGGGATCTCG-3′. The reactions were incubated at 95 °C for 5 min, followed by 40 cycles at 95 °C for 30 sec, 60 °C for 30 sec, and 72 °C for 30 sec. After the reactions were complete, the C_T_ values were determined using fixed threshold settings. The relative amount of PDCD4 mRNA was normalized to GAPDH.

### Profiling of miRNA expression

The expression profiles of miRNAs in gastric cancer tissues and corresponding noncancerous tissues was determined using the miRCURY LNA Array system (version. 18.0, Exiqon Inc., Woburn, MA, USA) and conducted by KangChen Bio-tech, Inc. (Shanghai, China). RNA samples were labeled using the miRCURY Hy3/Hy5 Power labeling kit (Exiqon Inc., Woburn, MA, USA) and hybridized on the miRCURY LNA Array station. Scanning was performed with the Axon GenePix 4000B microarray scanner (Molecular Devices, LLC, Sunnyvale, CA, USA). GenePix Pro version 6.0 was used to read the raw data of the images. The intensity of the signal was calculated after background subtraction, and replicated spots in the same image were averaged to obtain the median intensity. The median normalization method was used to obtain normalized data (foreground minus background divided by median). The significance of the results was determined using fold change and *t*-tests. The threshold value for significance used to define upregulation or downregulation of miRNAs was a fold change >2 and *p*-value <0.05.

### Overexpression and knockdown of miR-93

Synthetic pre-miR-93, anti-miR-93 and scrambled negative control RNAs (pre-miR-control and anti-miR-control) were purchased from Ambion (Austin, TX, USA). The cells were seeded onto 6-well plates or 60-mm dishes and transfected using Lipofectamine 2000 (Invitrogen) on the following day when the cells were approximately 70% confluent. In each well, equal amounts of pre-miR-93, anti-miR-93 or scrambled negative control RNA were used. The cells were harvested at 24 h after transfection for quantitative RT-PCR analysis and Western blotting.

### Luciferase reporter assay

To examine the direct binding of miR-93 to the target gene PDCD4, a luciferase reporter assay was performed as previously described^19^. The entire 3′-untranslated region (3′-UTR) of human PDCD4 was PCR amplified from human genomic DNA. The PCR products were inserted into the p-MIR-reporter plasmid (Ambion), and the insertion was confirmed through sequencing. To examine the binding specificity, the sequences that interacted with the miR-93 seed sequence were mutated (all two binding positions were mutated), and the mutant PDCD4 3′-UTR was inserted into an equivalent luciferase reporter. For luciferase reporter assays, AGS cells were cultured in 24-well plates, and each well was transfected with 1 μg of firefly luciferase reporter plasmid, 1 μg of a β-galactosidase (β-gal) expression plasmid (Ambion), and equal amounts (100 pmol) of pre-miR-93, anti-miR-93 or the scrambled negative control RNA using Lipofectamine 2000 (Invitrogen). The β-gal plasmid was used as a transfection control. Twenty-four hours after transfection, the cells were assayed using a luciferase assay kit (Promega, Madison, WI, USA).

### Plasmid construction and siRNA interference assay

The siRNA sequence targeting the human PDCD4 cDNA was designed and synthesized at GenePharma (Shanghai, China). The siRNA sequence was 5′-GCUGCUUUGGACAAGGCUATT-3′. A scrambled siRNA was included as a negative control. A mammalian expression plasmid encoding the human PDCD4 open reading frame (pReceiver-M02- PDCD4) was purchased from GeneCopoeia (Germantown, MD, USA). An empty plasmid served as a negative control. In addition, a miR-93 sponge was constructed by inserting six miR-93 complementary sites (in tandem) into the 3′ end of a non-coding RNA driven by the CMV promoter. A control sponge was constructed with six blank binding sites that are not complementary to any known miRNAs. The PDCD4 expression plasmid, miR-93 sponge plasmid and PDCD4 siRNA were transfected into AGS cells using Lipofectamine 2000 (Invitrogen) according to the manufacturer’s instructions. Total RNA and protein were isolated at 24 h post-transfection. The PDCD4 mRNA and protein expression levels were assessed through quantitative RT-PCR analysis and Western blotting.

### Protein extraction and Western blotting

All cells were rinsed with PBS (pH 7.4) and lysed on ice for 30 min in RIPA Lysis buffer (Beyotime, China) supplemented with a Protease and Phosphatase Inhibitor Cocktail (Thermo Scientific 78440). The tissue samples were frozen solid with liquid nitrogen, ground into powder, and lysed on ice for 30 min in RIPA lysis buffer containing the Protease and Phosphatase Inhibitor Cocktail. When necessary, sonication was used to facilitate lysis. Cell lysates or tissue homogenates were centrifuged for 10 min (12000 g, 4 °C). The supernatant was collected, and the protein concentration was calculated using the Pierce BCA protein assay kit (Thermo Scientific, Rockford, IL, USA). The protein levels were analyzed via Western blotting using the corresponding antibodies. The protein levels were normalized after probing the same blots with a GAPDH antibody. The following antibodies were purchased from the corresponding sources: anti-PDCD4 (k4C1) (Santa Cruz Biotechnology sc-130545, Santa Cruz, CA, USA) and anti-GAPDH (Santa Cruz Biotechnology sc-365062, Santa Cruz, CA, USA). The protein bands were analyzed using ImageJ software.

### Apoptosis assays

The apoptosis of AGS cells was examined using an Annexin V-FITC/propidium iodide (PI) staining assay. AGS cells were cultured in 12-well plates and transfected with pre-miR-93, anti-miR-93, PDCD4 siRNA, or the PDCD4 overexpression plasmid to induce apoptosis. Pre-miR-control, anti-miR-control, control siRNA, and control plasmids served as negative controls. The cells were cultured overnight in both serum-containing complete medium and serum-depleted medium, and the attached and floating cells were subsequently harvested. The apoptotic cells were identified through flow cytometry using an Annexin V-FITC/PI staining kit (BD Biosciences, CA, USA). After washing with cold PBS, the cells were re-suspended in binding buffer (100 mM HEPES, pH 7.4, 100 mM NaCl, and 25 mM CaCl_2_) followed by staining with Annexin V-FITC/PI at room temperature for 15 min in the dark. Apoptotic cells were subsequently evaluated by gating PI and Annexin V-positive cells on a fluorescence-activated cell-sorting (FACS) flow cytometer (BD Biosciences, San Jose, CA). All experiments were performed in triplicate.

Furthermore, nuclei were stained with DAPI to evaluate the morphological changes in apoptotic cells. Briefly, after transfection and culture for 24 h in serum-depleted medium, AGS cells were stained with DAPI for 15 min at 37 °C under 5% (v/v) CO_2_. After washing with cold PBS and fixing in 4% (v/v) formalin, cells were examined using an Olympus IX81 microscope (200×) equipped with X-CITE fluorescence illumination (Series 120Q; blue fluorescence). Moreover, TUNEL assay using DeadEnd Fluorometric TUNEL System kit was performed according to the manufacturer’s protocol (Promega, Mannheim, Germany). Results of DAPI staining and TUNEL staining were shown as representative of three independent experiments.

### Establishment of tumor xenografts in mice

Six-week-old male SCID (severe combined immune deficiency) mice (nu/nu) were purchased from the Model Animal Research Center of Nanjing University (Nanjing, China) and maintained under specific pathogen-free conditions at Nanjing University. AGS cells were infected with a control lentivirus or a miR-93 overexpression lentivirus, or transfected with a PDCD4 overexpression plasmid, or co-transfected with a miR-93 overexpression lentivirus and a PDCD4 overexpression plasmid. After infection and transfection, AGS cells were injected subcutaneously into xenograft mice (2 × 10^6^ cells per mouse, 5 mice per group). Mice were sacrificed after 60 days. The tumor xenografts were removed, and the weight of the tumors was measured. Parts of the tissues were used for protein and total RNA extraction, and the remains were fixed in 4% paraformaldehyde for 24 h and then processed for Hematoxylin and eosin (H&E) staining or immunohistochemical staining for PDCD4 and Ki-67.

### Statistical analysis

All Western blot images are representative of at least three independent experiments. Quantitative RT-PCR, luciferase reporter assays, and cell apoptosis assays were performed in triplicate, and each experiment was repeated several times. The data are shown as the means ± SE of at least three independent experiments. The differences were considered statistically significant at p < 0.05 using Student’s *t*-test.

## Results

### The downregulation of PDCD4 protein but not mRNA in gastric cancer tissues

**Figure 1:**
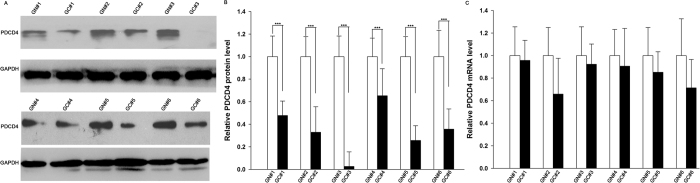
PDCD4 protein and mRNA expression levels in gastric cancer tissues. **(A,B)** Western blot analysis of the expression levels of the PDCD4 protein in 6 pairs of gastric cancer (GC) and normal adjacent (GN) tissue samples. (**A**) representative image; (**B**) quantitative analysis. **(C)** Quantitative RT-PCR analysis of the relative expression levels of PDCD4 mRNA in 6 pairs of GC and GN samples. ***P < 0.001.

We first measured the levels of PDCD4 protein in 6 pairs of gastric cancer tissues and corresponding noncancerous tissues (the clinical features of these tissue samples are listed in Supplementary Table 1). We observed that PDCD4 protein levels were significantly lower in the cancer tissues (Fig. 1A,B). Subsequently, we performed quantitative RT-PCR to measure the levels of PDCD4 mRNA in the same 6 pairs of cancerous and noncancerous tissues. However, PDCD4 mRNA levels did not significantly differ between cancerous and noncancerous tissues (Fig. 1C). The disparity between PDCD4 protein and mRNA levels in gastric cancer tissues suggested a post-transcriptional mechanism involved in the regulation of PDCD4.

### Identification of conserved miR-93 target sites within the 3′-UTR of PDCD4

**Figure 2:**
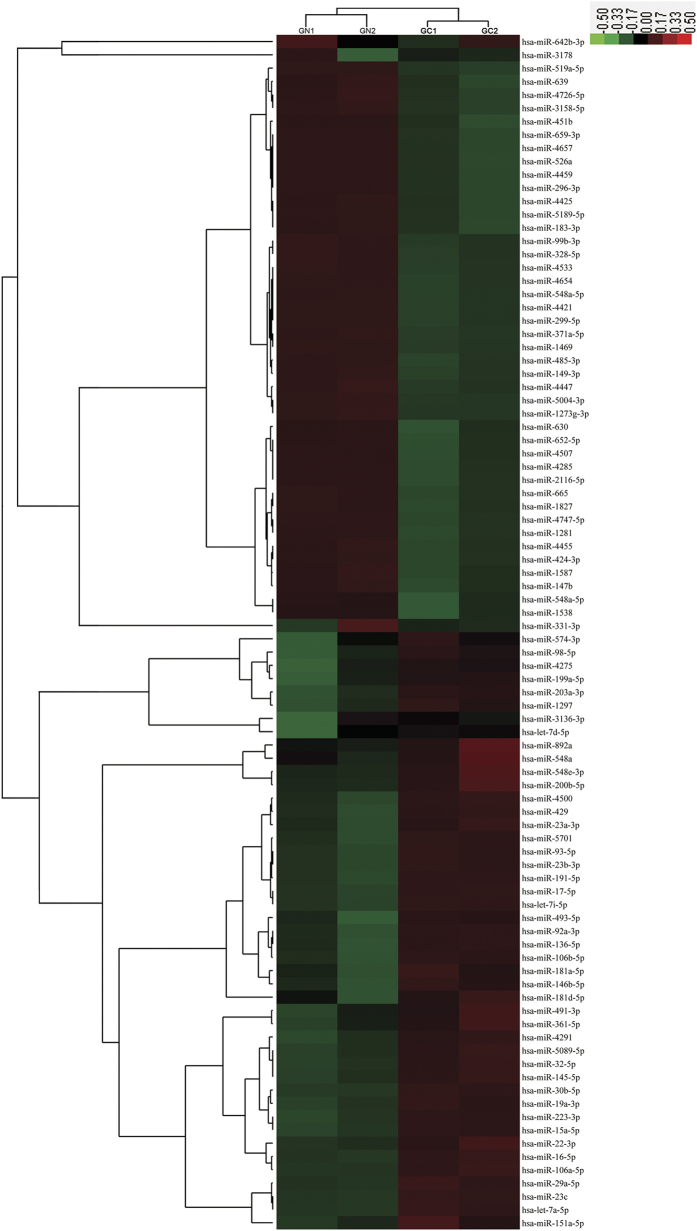
Profiling of miRNA expression in gastric cancer tissues and normal adjacent tissues by miRNA microarray technology. The expression of miRNAs is hierarchically clustered on the y axis, and GC (gastric cancer tissue samples) or GN (normal adjacent tissue samples) are hierarchically clustered on the x axis. The relative miRNA expression is depicted according to the color scale shown on the right. Red indicates upregulation; green, downregulation.

**Figure 3:**
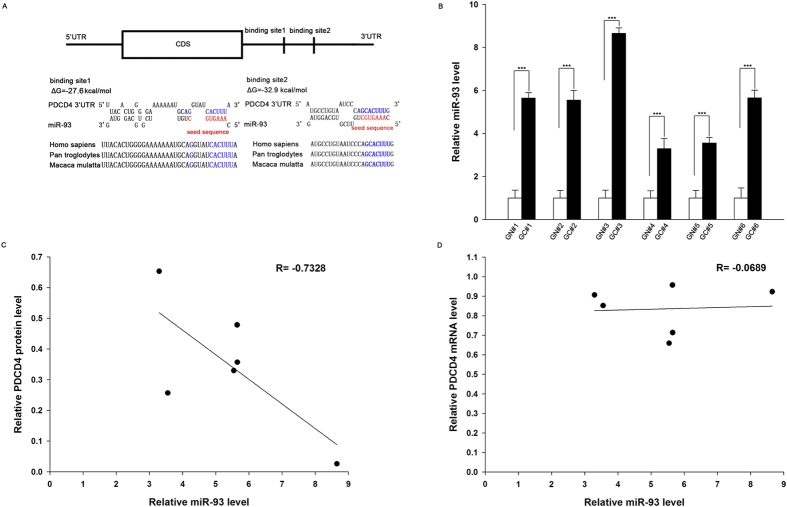
Detection of an inverse correlation between miR-93 and PDCD4 levels in gastric cancer tissue samples. **(A)** Schematic depicting the hypothetical duplexes formed through interactions between the binding sites in the PDCD4 3′-UTR (top) and miR-93 (bottom). The predicted free energy of each hybrid is indicated. The seed recognition sites are denoted, and all nucleotides in these regions are highly conserved across species. **(B)** Quantitative RT-PCR analysis of the miR-93 expression levels in six pairs of GC and GN samples. **(C)** Pearson’s correlation scatter plot of the fold-change in the levels of miR-93 and PDCD4 protein in human gastric cancer tissues. **(D)** Pearson’s correlation scatter plot of the fold-change in the levels of miR-93 and PDCD4 mRNA in human gastric cancer tissues. ***P < 0.001.

One important mode of post-transcriptional regulation is the repression of mRNA transcripts through miRNAs. To identify aberrantly expressed miRNAs that can potentially target PDCD4 in gastric cancer, a miRNA microarray was performed in two pairs of gastric cancer tissues and corresponding noncancerous tissues using the Exiqon miRCURY LNA microRNA Array. The levels of miRNAs differed significantly between gastric cancer tissues and corresponding noncancerous tissues (Fig. 2). Of the 1700 miRNAs detected on the microarray, 46 were found to be significantly upregulated and 44 were downregulated in the gastric cancer tissues compared with the corresponding noncancerous tissues (fold change >2 and *p*-value <0.05). Then, three computational algorithms, including TargetScan^20^, miRanda^8^ and PicTar^21^, were used in combination to investigate if the aberrantly expressed miRNAs could potentially target PDCD4. Among the 10 most upregulated miRNAs (Supplementary Table 2), miR-16-5p, miR-23b-3p, let-7a-5p, miR-15a-5p, miR-17-5p and miR-93 were identified as the candidate regulators of PDCD4. To determine whether these miRNAs could bind with the 3′-UTR of PDCD4 mRNA, the full-length 3′-UTR of PDCD4 was placed downstream of the firefly luciferase gene in a reporter plasmid. The resulting plasmid was transfected into the human gastric carcinoma cell line AGS along with pre-miRNAs of miR-16-5p, miR-23b-3p, let-7a-5p, miR-15a-5p, miR-17-5p and miR-93. In these experiments, pre-miRNAs were synthetic RNA oligonucleotides mimicking the miRNA precursors. As expected, cellular miRNA levels were indeed increased after transfection (Supplementary Fig. 1A). As a result, luciferase activity was markedly reduced in cells transfected with pre-miR-23b-3p, pre-miR-17-5p or pre-miR-93, while pre-miR-16-5p, pre-let-7a-5p and pre-miR-15a-5p have no influence on the luciferase activity (Supplementary Fig. 1B). These results suggested that miR-23b-3p, miR-17-5p and miR-93 could potentially bind to the 3′-UTR of PDCD4 mRNA transcript. Because the luciferase activity was reduced to maximal 80% of the original activity by miR-93, we next focused on miR-93. The predicted interaction between miR-93 and the target sites in the PDCD4 3′-UTR are illustrated in Fig. 3A. Two predicted hybridizations were identified between miR-93 and the 3′-UTR of PDCD4. The minimum free energy values of the two hybridizations were −27.6 and −32.9 kcal/mol, and these values are well within the range of genuine miRNA-target pairs. Furthermore, the miR-93 binding sequences in the PDCD4 3′-UTR are highly conserved across species.

### Detection of an inverse correlation between miR-93 and PDCD4 levels in gastric cancer tissues

We next investigated whether miR-93 was inversely correlated with PDCD4 in gastric cancer. After determining the levels of miR-93 in the same 6 pairs of gastric cancer tissues and noncancerous tissues, we observed that miR-93 levels were indeed upregulated in gastric cancer tissues (Fig. 3B). The inverse correlation between miR-93 and PDCD4 protein levels (Fig. 3C) and the disparity between the miR-93 and PDCD4 mRNA levels (Fig. 3D) was further illustrated using Pearson’s correlation scatter plots. Because animal miRNAs generally block translational processes without affecting transcript levels, these results implied the involvement of a miR-93-mediated post-transcriptional regulatory mechanism in PDCD4 repression.

### Validation of PDCD4 as a direct target of miR-93

**Figure 4:**
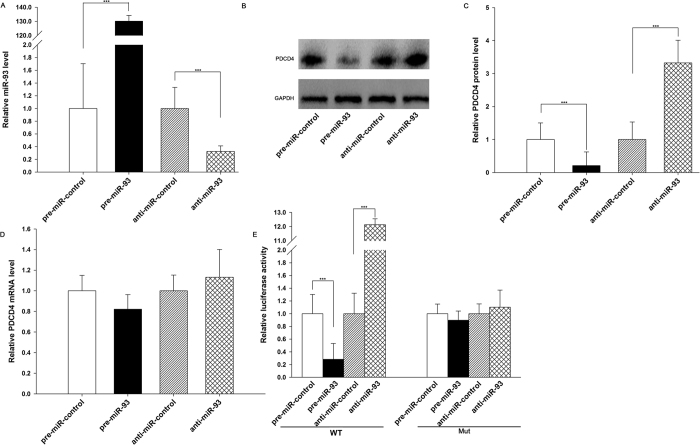
Direct post-transcriptional regulation of PDCD4 expression through miR-93. **(A)** Quantitative RT-PCR analysis of the miR-93 levels in AGS cells treated with pre-miR-control, pre-miR-93, anti-miR-control or anti-miR-93. **(B,C)** Western blot analysis of PDCD4 protein levels in AGS cells treated with pre-miR-control, pre-miR-93, anti-miR-control or anti-miR-93. (**B**) representative image; (**C**) quantitative analysis. **(D)** Quantitative RT-PCR analysis of PDCD4 mRNA levels in AGS cells treated with pre-miR-control, pre-miR-93, anti-miR-control or anti-miR-93. **(E)** Firefly luciferase reporters containing either wild-type (WT) or mutant (Mut) miR-93 binding sites in the PDCD4 3′-UTR were co-transfected into AGS cells along with pre-miR-control, pre-miR-93, anti-miR-control or anti-miR-93. Twenty-four hours post-transfection, the cells were assayed using a luciferase assay kit. ***P < 0.001.

The correlation between miR-93 and PDCD4 was further examined after evaluating PDCD4 expression in AGS cells after the overexpression or knockdown of miR-93. Knockdown of miR-93 was achieved after transfecting cells with anti-miR-93, a chemically modified antisense oligonucleotide designed to specifically target mature miR-93. The efficient overexpression and knockdown of miR-93 in AGS cells is shown in Fig. 4A. Cellular miR-93 levels were increased approximately 130-fold when AGS cells were transfected with pre-miR-93, and these levels decreased to approximately 30% of the normal level when AGS cells were treated with anti-miR-93. As anticipated, overexpressing miR-93 significantly suppressed the PDCD4 protein levels in AGS cells, whereas miR-93 knockdown had the opposite effect on PDCD4 expression in these cells (Fig. 4B,C). To determine the regulatory level at which miR-93 influenced PDCD4 expression, we repeated the above experiments and examined the expression of PDCD4 mRNA after transfection. Neither the overexpression nor the knockdown of miR-93 affected PDCD4 mRNA levels in AGS cells (Fig. 4D). Furthermore, because miRNA sponge technology has been developed as an efficient tool for miRNA silencing, we employed this technology instead of transfection with miRNA antisense to knock down miR-93 and prove the role of miR-93 in suppression of PDCD4 protein in AGS cells (Supplementary Fig. 2A). The miR-93 sponge efficiently knocked down miR-93 expression in AGS cells (Supplementary Fig. 2B). As a result, PDCD4 protein levels were significantly increased in AGS cells (Supplementary Fig. 2C,D).

To determine whether the negative regulatory effects of miR-93 on PDCD4 expression were mediated through the binding of miR-93 to the presumed sites in the 3′-UTR of the PDCD4 mRNA, the full-length 3′-UTR of PDCD4, containing the two presumed miR-93 binding sites, was placed downstream of the firefly luciferase gene in a reporter plasmid. The resulting plasmid was transfected into AGS cells along with pre-miR-93, anti-miR-93 or scrambled negative control RNAs. As expected, luciferase activity was markedly reduced in cells transfected with pre-miR-93 and increased in the cells transfected with anti-miR-93 (Fig. 4E). Furthermore, we introduced point mutations into the corresponding complementary sites in the 3′-UTR of PDCD4 to eliminate the predicted miR-93 binding sites (all two binding positions were mutated). This mutated luciferase reporter was unaffected through either the overexpression or knockdown of miR-93 (Fig. 4E). This finding suggested that the binding sites strongly contribute to the interaction between miR-93 and PDCD4 mRNA. In summary, these results demonstrated that miR-93 directly binds to the 3′-UTR of the PDCD4 mRNA transcript and inhibits PDCD4 translation in gastric cancer cells.

### The role of miR-93 in regulating PDCD4 in gastric cancer cells

**Figure 5:**
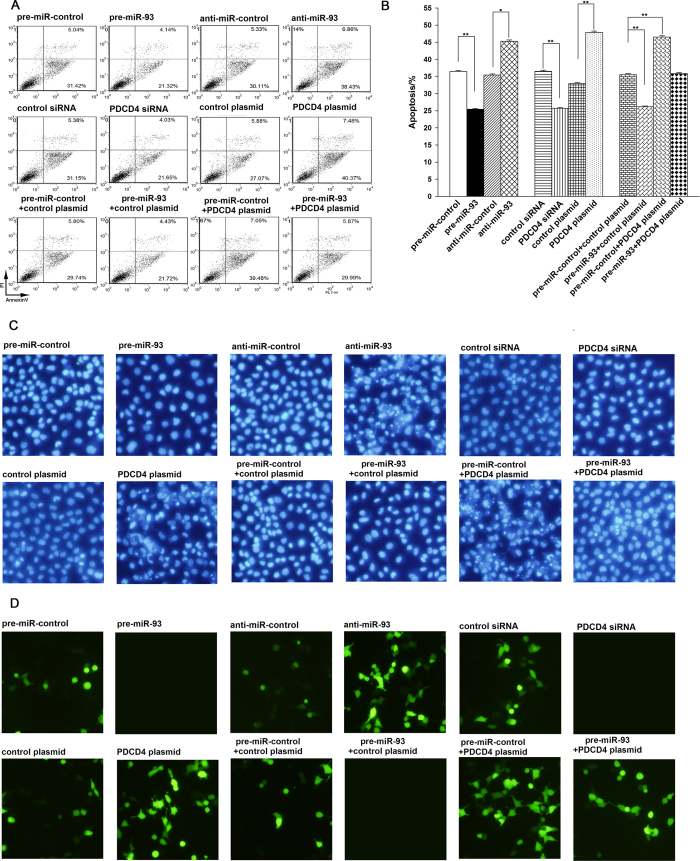
The role of PDCD4 targeting through miR-93 in the regulation of apoptosis in gastric cancer cells. AGS cells were transfected with equal doses of pre-miR-control, pre-miR-93, anti-miR-control, anti-miR-93, control siRNA, PDCD4 siRNA, control plasmid, PDCD4 plasmid, pre-miR-control plus control plasmid, pre-miR-93 plus control plasmid, pre-miR-control plus PDCD4 plasmid, or pre-miR-93 plus PDCD4 plasmid. **(A,B)** The cell apoptosis profiles were analyzed using Annexin V-FITC/PI staining in flow cytometry. The biparametric histogram shows cells in early (bottom right quadrant) and late apoptotic states (upper right quadrant). Viable cells are double negative (bottom left quadrant). (**A**) representative image; (**B**) quantitative analysis. **(C)** Representative images of apoptotic AGS cells analyzed using DAPI staining. DAPI staining is used to monitor the apoptosis of cells by exhibiting a strong fluorescence when DAPI becomes bound to natural double-stranded DNA. The normal cell nuclei are round in shape and staining is evenly distributed. When the cells become apoptotic, the cell nuclei become deformed due to the aggregation of the DNA. (**D**) Representative images of apoptotic AGS cells analyzed using TUNEL staining. TUNEL staining is used to monitor the apoptosis of cells by exhibiting a strong fluorescence when DNA strand breaks in the apoptotic cells and is labeled with fluorescein-12-dUTP. *P < 0.05; **P < 0.01.

We next analyzed the biological consequences of the miR-93-driven repression of PDCD4 expression in gastric cancer cells. Because PDCD4 is involved in the regulation of cell apoptosis, we evaluated the effects of miR-93 on the apoptosis of AGS cells using Annexin V-FITC/PI staining, DAPI staining and TUNEL staining. As expected, AGS cells transfected with pre-miR-93 showed decreased apoptosis; in contrast, knocking down miR-93 with anti-miR-93 had the opposite effect on cell apoptosis (Fig. 5). Moreover, knocking down miR-93 with the miR-93 sponge also increased apoptosis in AGS cells (Supplementary Fig. 2E–G).

Subsequently, we investigated whether the overexpression or knockdown of PDCD4 would impact cell apoptosis. To knock down PDCD4, a siRNA targeting PDCD4 was designed and transfected into AGS cells. To overexpress PDCD4, an expression plasmid designed to specifically express the full-length open reading frame (ORF) of PDCD4 without the miR-93-responsive 3′-UTR was constructed and transfected into AGS cells. The efficient knockdown and overexpression of PDCD4 in AGS cells is shown in Supplementary Fig. 3. AGS cells transfected with PDCD4 siRNA showed decreased cell apoptosis; in contrast, transfection with the PDCD4-overexpression plasmid had the opposite effect on cell apoptosis (Fig. 5). Moreover, compared with cells transfected with pre-miR-93 alone, those transfected with both pre-miR-93 and the PDCD4-overexpression plasmid exhibited significantly higher apoptosis rates (Fig. 5), suggesting that miR-93-resistant PDCD4 is sufficient to rescue the suppression of PDCD4 through miR-93 and attenuate the anti-apoptosis effect of miR-93 on gastric cancer cells. Taken together, these results indicate that miR-93 might inhibit cell apoptosis through silencing PDCD4.

### The influence of miR-93 and PDCD4 on the growth of gastric cancer cells *in vivo*

**Figure 6:**
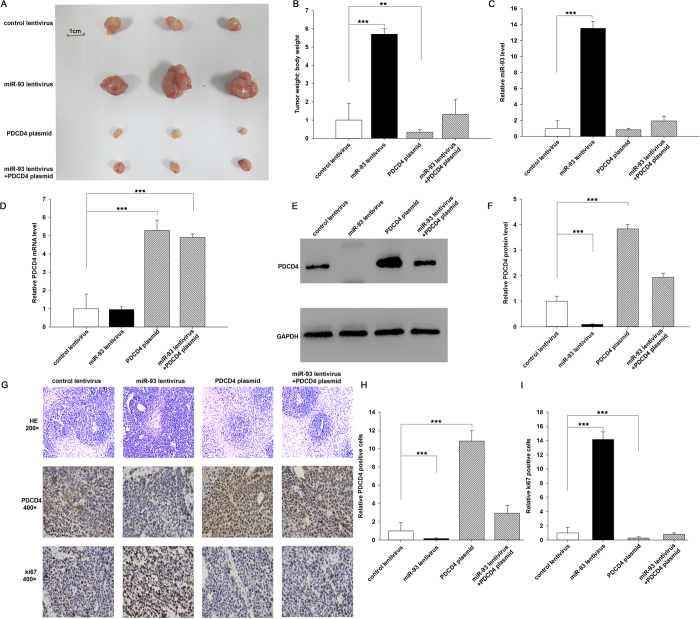
Effects of miR-93 and PDCD4 on the growth of gastric cancer cell xenografts in mice. AGS cells were infected with a control lentivirus or a miR-93 overexpression lentivirus, or transfected with a PDCD4 overexpression plasmid, or co-transfected with the miR-93 overexpression lentivirus plus PDCD4 overexpression plasmid. Then the cells (2 × 10^6^ cells per 0.1 mL) were implanted subcutaneously into 6-week-old SCID mice (5 mice per group), and tumor growth was evaluated at day 60 after cell implantation. **(A)** Representative images of the tumors from the implanted mice. **(B)** Quantitative analysis of the tumor weights. **(C)** Quantitative RT-PCR analysis of miR-93 levels in the tumors from implanted mice. **(D)** Quantitative RT-PCR analysis of PDCD4 mRNA levels in the tumors from implanted mice. **(E,F)** Western blotting analysis of PDCD4 protein levels in the tumors from implanted mice. (**E**) representative image; (**F**) quantitative analysis. **(G–I)** H & E-stained sections and immunohistochemical staining for PDCD4 and Ki-67 in the tumors from implanted mice. (**G**) representative image; (**H**,**I**) quantitative analysis. **p < 0.01; ***p < 0.001.

We evaluated the biological effects of miR-93 and PDCD4 on the growth of gastric cancer cells in a gastric cancer xenograft mouse model. A 300-bp fragment containing the genomic miR-93 sequence was cloned into a lentiviral expression plasmid, and AGS cells were infected with the lentiviral plasmid to express miR-93. The efficient overexpression of miR-93 and inhibition of PDCD4 protein in AGS cells by lentiviral transfection is shown in Supplementary Fig. 4. AGS cells were also transfected with a PDCD4 overexpression plasmid. Subsequently, AGS cells (2 × 10^6^ cells per 0.1 mL) were infected with the miR-93 overexpression lentivirus, or transfected with the PDCD4 overexpression plasmid, or co-transfected with the miR-93 overexpression lentivirus plus PDCD4 overexpression plasmid. Then the cells were implanted subcutaneously into 6-week-old SCID mice. After 60 days of xenograft growth *in vivo*, the mice were sacrificed, and the weight of the tumors was measured. A significant increase in the sizes and weights of the tumors was observed in the miR-93-overexpressing group compared to the control group, whereas the sizes and weights of the tumors in the group implanted with the PDCD4-overexpression plasmid were dramatically decreased (Fig. 6A,B). Additionally, PDCD4 overexpression attenuated the promotive effect of miR-93 on tumor growth (Fig. 6A,B), suggesting that miR-93 might promote tumor growth by silencing PDCD4. Subsequently, total RNA and protein were isolated from the tumors and analyzed. After 60 days of xenograft growth *in vivo*, tumors from the miR-93-overexpression group showed a significant increase in the expression of miR-93 compared to tumors from the control group (Fig. 6C). Likewise, PDCD4 mRNA levels were increased in the tumors from the PDCD4-overexpressing group (Fig. 6D). Tumors from the miR-93-overexpressing group displayed reduced PDCD4 protein levels compared to tumors from the control group, whereas the tumors from the PDCD4-overexpressing group showed elevated PDCD4 protein levels (Fig. 6E,F). Tumors with both miR-93 and PDCD4 overexpression exhibited significantly higher levels of PDCD4 compared to tumors with miR-93 overexpression (Fig. 6E,F), suggesting that PDCD4 overexpression could rescue the PDCD4 suppression caused by miR-93. Furthermore, Hematoxylin and eosin (H & E) staining of xenograft tissues showed more cell mitosis in the group implanted with the miR-93 lentivirus compared with the control group, whereas confluent necrotic areas were observed in xenografts from the PDCD4-overexpressing group (Fig. 6G). Xenografts with both miR-93 and PDCD4 overexpression exhibited reduced cell mitosis compared to xenografts with miR-93 overexpression (Fig. 6G), suggesting that PDCD4 overexpression could attenuate the anti-apoptosis effect of miR-93. Immunohistochemical staining also revealed the presence of lower levels of PDCD4 in the tumors from mice implanted with miR-93-overexpressing cells, whereas the tumors from the PDCD4-overexpressing mice showed increased PDCD4 protein levels (Fig. 6G,H). Finally, the proliferative activity of tumor cells was assessed by immunocytochemistry with the mouse monoclonal antibody Ki-67. The cell proliferation rate measured by the percentage of Ki-67-positive tumor cells was decreased in the group implanted with the PDCD4 overexpression plasmid and increased in the group implanted with the miR-93 overexpression lentivirus (Fig. 6G,I). Likewise, PDCD4 overexpression attenuated the anti-apoptosis effect caused by miR-93 overexpression (Fig. 6G,I). These results were consistent with the findings of the *in vitro* assays, which firmly validated the role of miR-93 in promoting tumorigenesis through the targeting of PDCD4.

## Discussion

Gastric cancer is one of the most common cancers and leading causes of cancer death worldwide. Many genes, including cancer inhibitors (tumor suppressors) and cancer inducers (oncogenes), influence gastric carcinogenesis. PDCD4 plays a pivotal role in the occurrence and development of gastric cancer. Wang *et al.* confirmed that PDCD4 mediates the sensitivity of gastric cancer cells to apoptosis by downregulation of FLIP, a negative regulator of apoptosis^5^. Eto *et al.* showed that PDCD4 is a critical suppressor of apoptosis by inhibiting the translation of procaspase-3 mRNA^22^. These findings indicate that PDCD4 participates in tumorigenesis through the regulation of apoptosis. In the present study, we observed that silencing PDCD4 expression through siRNA inhibits apoptosis in gastric cancer cells, whereas overexpressing PDCD4 induced the opposite effects, validating a role for this protein as an essential anti-oncogene during gastric tumorigenesis. Interestingly, we identified a discordance between PDCD4 protein and mRNA levels in human gastric cancer tissues. These results suggest a post-transcriptional regulation mechanism in PDCD4 repression. One centrally important mode of post-transcriptional regulation is the repression of mRNA transcripts through miRNAs. Therefore, we searched for miRNAs that target PDCD4 and identified miR-93 as a candidate. In addition, after overexpressing or knocking down miR-93 in gastric cancer cells, we experimentally validated the direct inhibition of PDCD4 translation through miR-93. Moreover, we showed that miR-93 inhibited PDCD4 expression and consequently inhibited apoptosis in cultured gastric cancer cells. These results indicate the importance of miR-93 targeting PDCD4 as a novel regulatory pathway in gastric cancer progression.

miRNAs are aberrantly expressed in cancer and function as oncogenes or tumor suppressor genes^23^,^24^. In the present study, we observed that the levels of miR-93 were higher in gastric cancer tissues than in noncancerous tissues. These results suggest that miR-93 might be involved in the pathogenesis of gastric cancer as an oncomiR. Indeed, miR-93, derived from a paralog (miR-106b-25) of the oncogenic miR-17–92 cluster, is overexpressed in several types of cancers, including gastric cancer^10^, lung cancer^11^, breast cancer^17^ and hepatocellular carcinoma^25^. Furthermore, miR-93 plays an oncogenic role in cancer through the regulation of cell survival, tumor growth, apoptosis, cell-cycle distribution, migration and angiogenesis^11^,^26^,^27^,^28^,^29^. In the present study, we observed that overexpressing miR-93 inhibits apoptosis in gastric cancer cells, and reducing PDCD4 expression mimics miR-93 induction. Interestingly, we observed that the restoration of PDCD4 expression successfully attenuates the anti-apoptotic effects of miR-93 on gastric cancer cells, although miR-93 has many other targets. These results suggest that the targeting of PDCD4 is a major mechanism through which miR-93 exerts a tumor-promotive function.

Taken as a whole, this study delineates a novel regulatory network employing miR-93 and PDCD4 to fine-tune apoptosis in gastric cancer cells. This study might open new avenues for future gastric cancer therapies.

## Additional Information

**How to cite this article**: Liang, H. *et al.* miR-93 functions as an oncomiR for the downregulation of PDCD4 in gastric carcinoma. *Sci. Rep.* **6**, 23772; doi: 10.1038/srep23772 (2016).

## Supplementary Material

Supplementary Information
