# Author Correction: miR-93 functions as an oncomiR for the downregulation of PDCD4 in gastric carcinoma

**DOI:** 10.1038/s41598-021-98187-2

**Published:** 2021-09-14

**Authors:** Hongwei Liang, Feng Wang, Danping Chu, Weijie Zhang, Zhicong Liao, Zheng Fu, Xin Yan, Hao Zhu, Wen Guo, Yujing Zhang, Wenxian Guan, Xi Chen

**Affiliations:** 1 State Key Laboratory of Pharmaceutical Biotechnology, NJU Advanced Institute for Life Sciences, Jiangsu Engineering Research Center for MicroRNA Biology and Biotechnology, School of Life Science, Nanjing University, Nanjing, 210093 Jiangsu China; 2 Department of General Surgery, The Affiliated Drum Tower Hospital of Medical School of Nanjing University and Nanjing Multi-Center Biobank, Nanjing, 210008 Jiangsu China; 3 Department of Cardio-Thoracic Surgery, The Affiliated Drum Tower Hospital of Medical School of Nanjing University and Nanjing Multi-Center Biobank, Nanjing, 210008 Jiangsu China; 4 Department of Respiratory Medicine, The Affiliated Drum Tower Hospital of Medical School of Nanjing University and Nanjing Multi-Center Biobank, Nanjing, 210008 Jiangsu China; 5 Department of Gastroenterology, The Affiliated Drum Tower Hospital of Medical School of Nanjing University and Nanjing Multi-Center Biobank, Nanjing, 210008 Jiangsu China; 6 Department of Endocrinology, Nanjing Municipal Hospital for Governmental Organizations, Nanjing, 210018 Jiangsu China

Correction to: *Scientific Reports* 10.1038/srep23772, published online 29 March 2016

The original version of this Article contains errors in Figure 6G.

The image showing miR-93 lentivirus + PDCD4 plasmid at HE 200× is a partial duplication of the image showing control lentivirus at HE 200× .

The correct Figure 6 and accompanying legend appear below.

**Figure 6:**
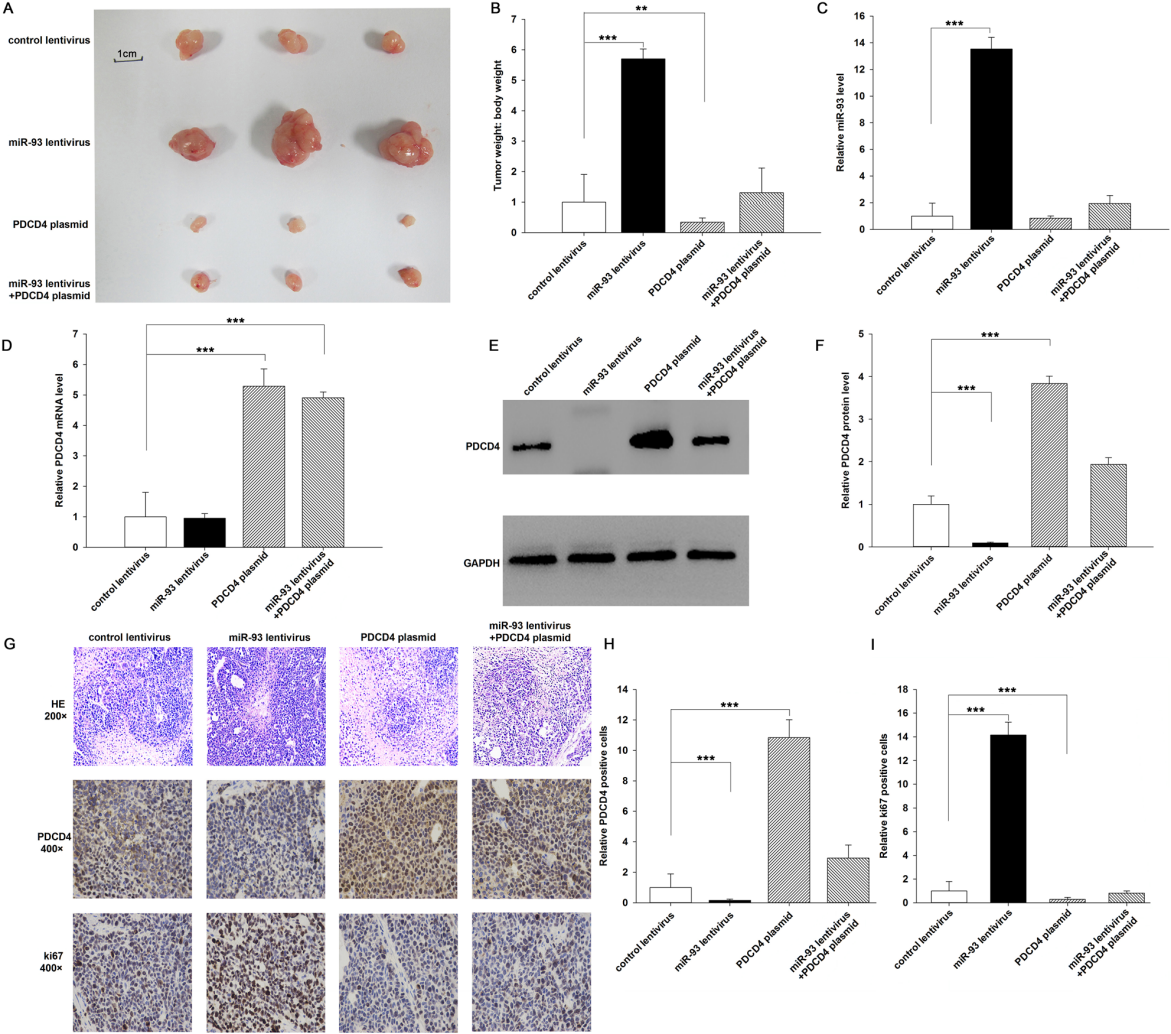
Effects of miR-93 and PDCD4 on the growth of gastric cancer cell xenografts in mice. AGS cells were infected with a control lentivirus or a miR-93 overexpression lentivirus, or transfected with a PDCD4 overexpression plasmid, or co-transfected with the miR-93 overexpression lentivirus plus PDCD4 overexpression plasmid. Then the cells (2 × 10^6^ cells per 0.1 mL) were implanted subcutaneously into 6-week-old SCID mice (5 mice per group) and tumor growth was evaluated at day 60 after cell implantation. **(A)** Representative images of the tumors from the implanted mice. **(B)** Quantitative analysis of the tumor weights. **(C)** Quantitative RT-PCR analysis of miR-93 levels in the tumors from implanted mice. **(D)** Quantitative RT-PCR analysis of PDCD4 mRNA levels in the tumors from implanted mice. **(E,F)** Western blotting analysis of PDCD4 protein levels in the tumors from implanted mice. (**E**) representative image; (**F**) quantitative analysis. **(G–I)** H & E-stained sections and immunohistochemical staining for PDCD4 and Ki-67 in the tumors from implanted mice. (**G**) representative image; (**H**,**I**) quantitative analysis. **p < 0.01; ***p < 0.001.

