# Supplementary material for: miR-93 functions as an oncomiR for the downregulation of PDCD4 in gastric carcinoma
Source: Sci Rep. 2016 Mar 29;6:23772. doi: 10.1038/srep23772 (PMC4810498; doi:10.1038/srep23772)

**miR-93 functions as an oncomiR for the downregulation of PDCD4 in gastric carcinoma**

**Supplementary Materials**

Hongwei Liang^1,*^, Feng Wang^2,*^, Danping Chu^1,*^, Weijie Zhang^2,*^, Zhicong Liao^3,*^, Zheng Fu^1^, Xin Yan^4^, Hao Zhu^5^, Wen Guo^6^, Yujing Zhang^1,#^, Wenxian Guan^2,#^, Xi Chen^1,#^

^1^State Key Laboratory of Pharmaceutical Biotechnology, NJU Advanced Institute for Life Sciences, Jiangsu Engineering Research Center for MicroRNA Biology and Biotechnology, School of Life Science, Nanjing University, Nanjing, Jiangsu 210093, China; ^2^Department of General Surgery, ^3^Department of Cardio-Thoracic Surgery, ^4^Department of Respiratory Medicine, ^5^Department of Gastroenterology, The Affiliated Drum Tower Hospital of Medical 11 School of Nanjing University and Nanjing Multi-center Biobank, Nanjing, Jiangsu 210008, China; ^6^Department of Endocrinology, Nanjing Municipal Hospital for Governmental Organizations, Nanjing, Jiangsu 210018, China.

*These authors contributed equally to this work.

#Correspondence: xichen@nju.edu.cn (Xi Chen), guan-wx@163.com (Wenxian Guan) or yjzhang@nju.edu.cn (Yujing Zhang)

**Supplementary Table 1. Demographic information of gastric carcinoma patient cohort.**

|  | **Gender** | **Age** | **Pathological Stage** | **HER2** | **VEGF** | **EGFR** | **MGMT** | **COX2** | **TS** | **Beta-tubulin** | **ERCC1** | **BRCA1** | **Ki67** |
| --- | --- | --- | --- | --- | --- | --- | --- | --- | --- | --- | --- | --- | --- |
| case#1 | Male | 54 | IB(T2,No,cMo) | - | + | + | + | +++ | - | ++ | + | + | 60% |
| case#2 | Male | 73 | IB(T2,No,cMo) | ++ | +++ | + | +++ | +++ | + | +++ | - | ++ | 80% |
| case#3 | Female | 71 | IV(T3,N1,M1) | - | + | - | +++ | ++ | - | +++ | + | - | 30% |
| case#4 | Female | 63 | IIIC(T4a,N3b,cMo) | - | + | - | +++ | +++ | + | +++ | + | ++ | 70% |
| case#5 | Male | 56 | IIIA(T3,N1,cMo) | ++ | ++ | - | +++ | +++ | + | +++ | + | ++ | 60% |
| case#6 | Female | 61 | IIA(T3,No,cMo) | + | + | - | + | +++ | - | ++ | - | - | 30% |

**Supplementary** [**Table 2**](http://www.ncbi.nlm.nih.gov/pmc/articles/PMC4505325/table/t1/)**. Significantly upregulated miRNAs in the gastric cancer tissues compared with the corresponding noncancerous tissues measured by miRNA microarray.**

| **miRNA** | **Fold change** | ***P* value** |
| --- | --- | --- |
| miR-16-5p | 72.80 | 0.04 |
| miR-23b-3p | 68.96 | 0.01 |
| let-7a-5p | 65.60 | 0.03 |
| miR-15a-5p | 53.59 | 0.00 |
| miR-17-5p | 33.59 | 0.00 |
| miR-19a-3p | 32.85 | 0.04 |
| miR-30b-5p | 31.31 | 0.00 |
| miR-93-5p | 26.86 | 0.01 |
| miR-191-5p | 20.99 | 0.00 |
| miR-32-5p | 20.59 | 0.04 |

**Supplementary Figure 1.**

**Selection of miRNAs that can potentially target PDCD4 by firefly luciferase reporter assay. (A)** Quantitative RT-PCR analysis of the miRNA levels in AGS cells transfected with pre-miR-control, pre-miR-16-5p, pre-miR-23b-3p, pre-let-7a-5p, pre-miR-15a-5p, pre-miR-17-5p or pre-miR-93. **(B)** Firefly luciferase reporters containing the PDCD4 3’-UTR were co-transfected into AGS cells along with pre-miR-control, pre-miR-16-5p, pre-miR-23b-3p, pre-let-7a-5p, pre-miR-15a-5p, pre-miR-17-5p or pre-miR-93. Twenty-four hours post-transfection, the cells were assayed using a luciferase assay kit. * P < 0.05; *** P < 0.001.


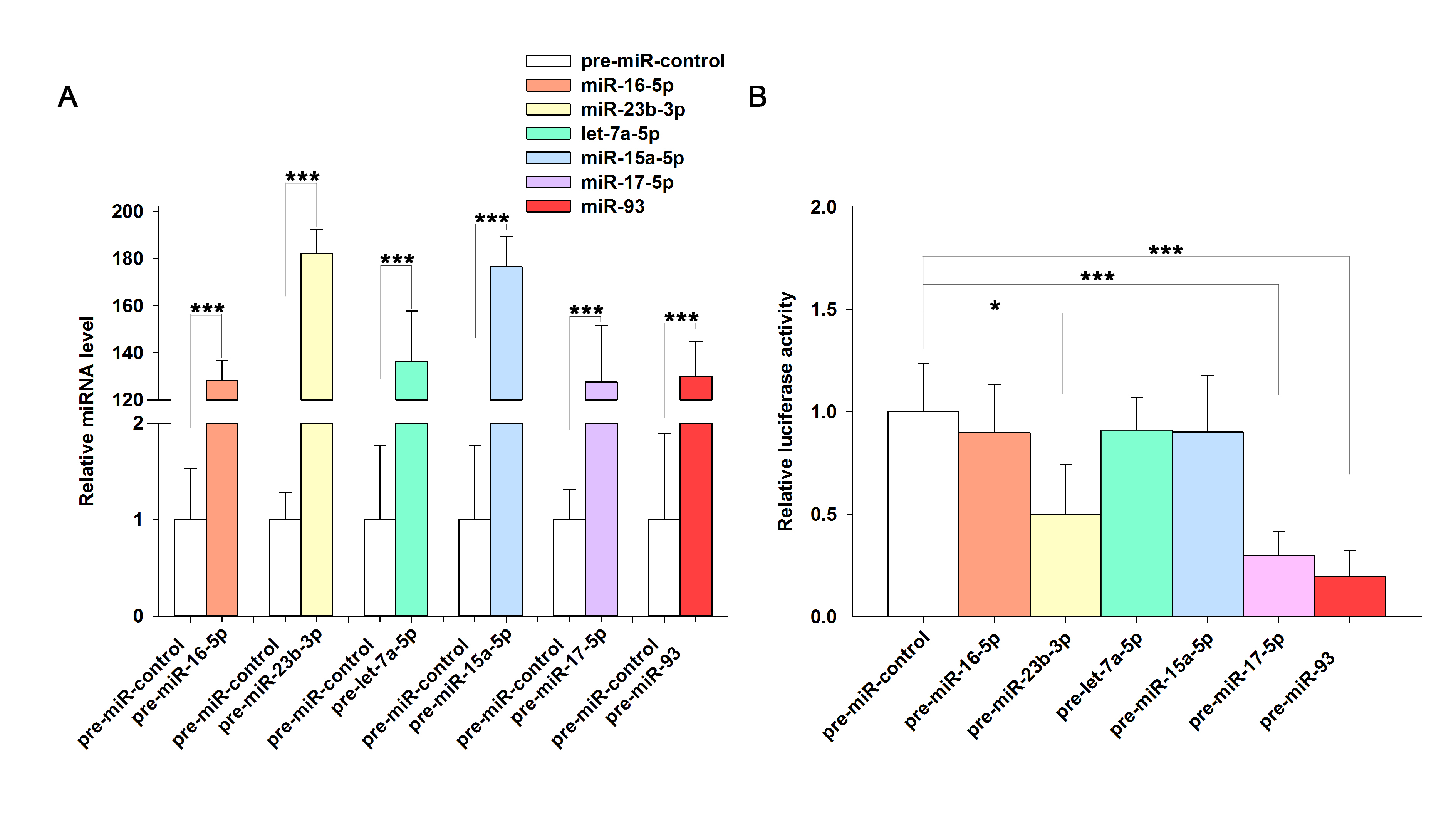


**Supplementary Figure 2. Knockdown of miR-93 expression in AGS cells by miRNA sponge technology. (A)** Design of the miR-93 sponge. A miR-93 sponge was constructed by inserting six miR-93 complementary sites (in tandem) into the 3’ end of a non-coding RNA driven by the CMV promoter. A control sponge was constructed with six blank binding sites that are not complementary to any known miRNAs. **(B)** Quantitative RT-PCR analysis of the miR-93 levels in AGS cells transfected with the control sponge or miR-93 sponge. **(C and D)** Western blot analysis of PDCD4 protein levels in AGS cells transfected with the control sponge or miR-93 sponge. C: representative image; D: quantitative analysis. **(E and F)** The cell apoptosis profiles were analyzed using flow cytometry. AGS cells were transfected with equal doses of control sponge or miR-93 sponge. The biparametric histogram shows cells in early (bottom right quadrant) and late apoptotic states (upper right quadrant). Viable cells are double negative (bottom left quadrant). E: representative image; F: quantitative analysis. **(G)** Representative images of apoptotic AGS cells analyzed using DAPI staining. AGS cells were transfected with equal doses of control sponge or miR-93 sponge. The extent of apoptosis was quantified by calculating the ratio of condensed nuclei to total nuclei. *** P < 0.001.


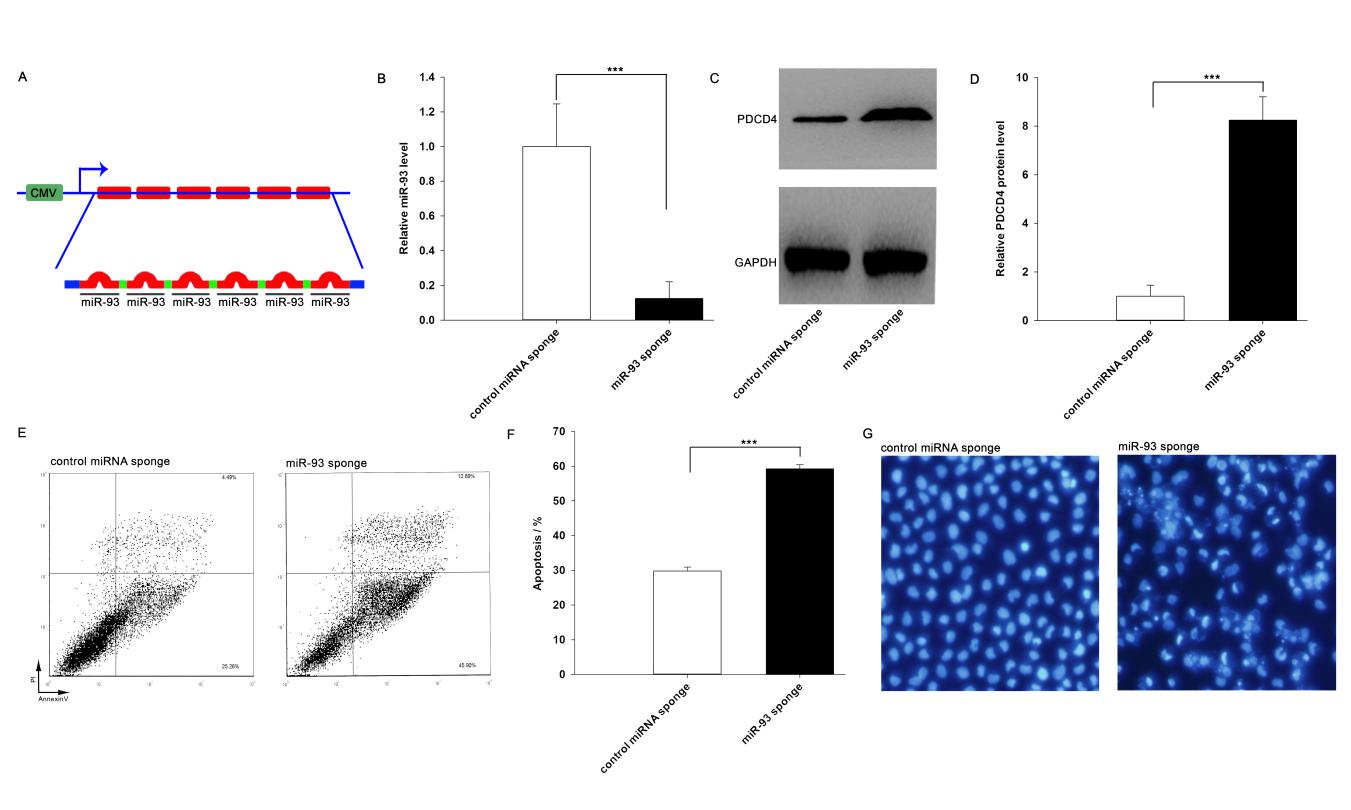


**Supplementary Figure 3. Downregulation of PDCD4 by siRNA and upregulation of PDCD4 by overexpression plasmid in gastric cancer cells.** **(A)** Quantitative RT-PCR analysis of PDCD4 mRNA levels in AGS cells treated with control siRNA, PDCD4 siRNA, control plasmid or PDCD4 plasmid. **(B and C)** Western blotting analysis of PDCD4 protein levels in AGS cells treated with control siRNA, PDCD4 siRNA, control plasmid or PDCD4 plasmid. B: representative image; C: quantitative analysis. ** P < 0.01; *** P < 0.001.


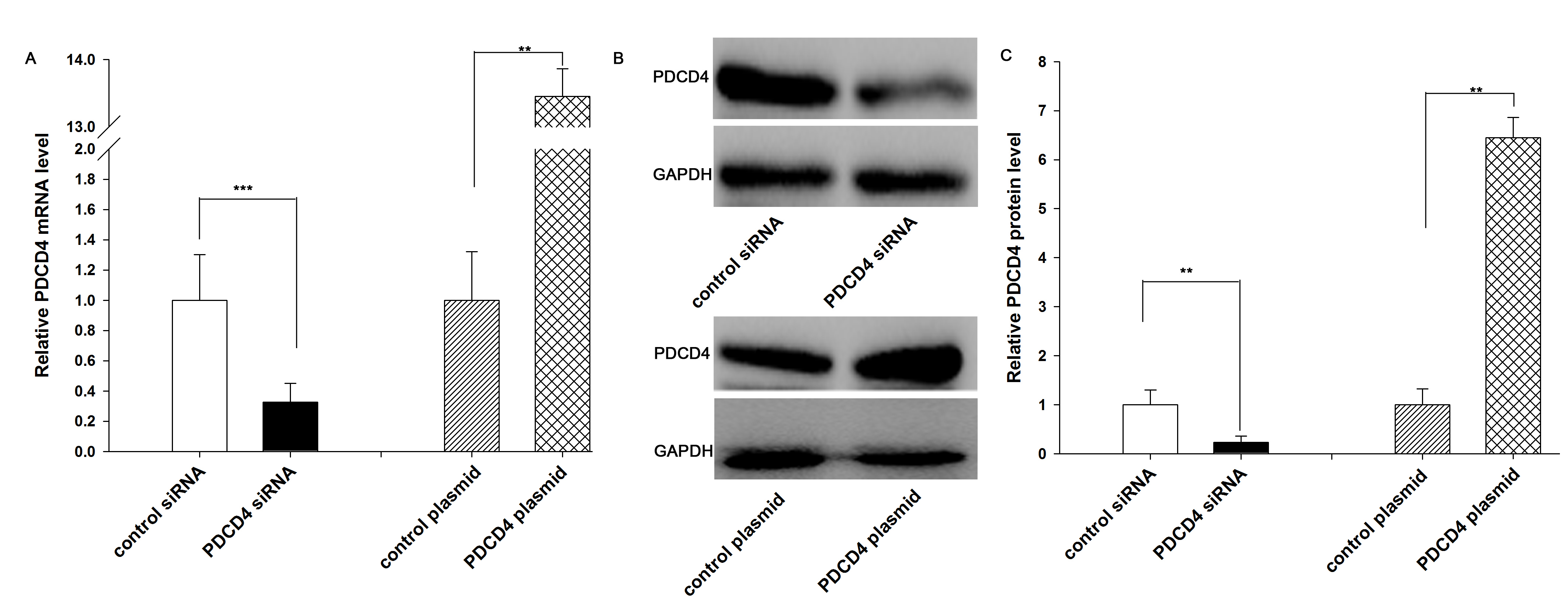


**Supplementary Figure 4. Upregulation of miR-93 by miR-93 lentivirus.** **(A)** Quantitative RT-PCR analysis of miR-93 levels in AGS cells treated with control lentivirus or miR-93 lentivirus. **(B)** Quantitative RT-PCR analysis of PDCD4 mRNA levels in AGS cells treated with control lentivirus or miR-93 lentivirus. **(C and D)** Western blotting analysis of PDCD4 protein levels in AGS cells treated with control lentivirus or miR-93 lentivirus. C: representative image; D: quantitative analysis. ** P < 0.01; *** P < 0.001.


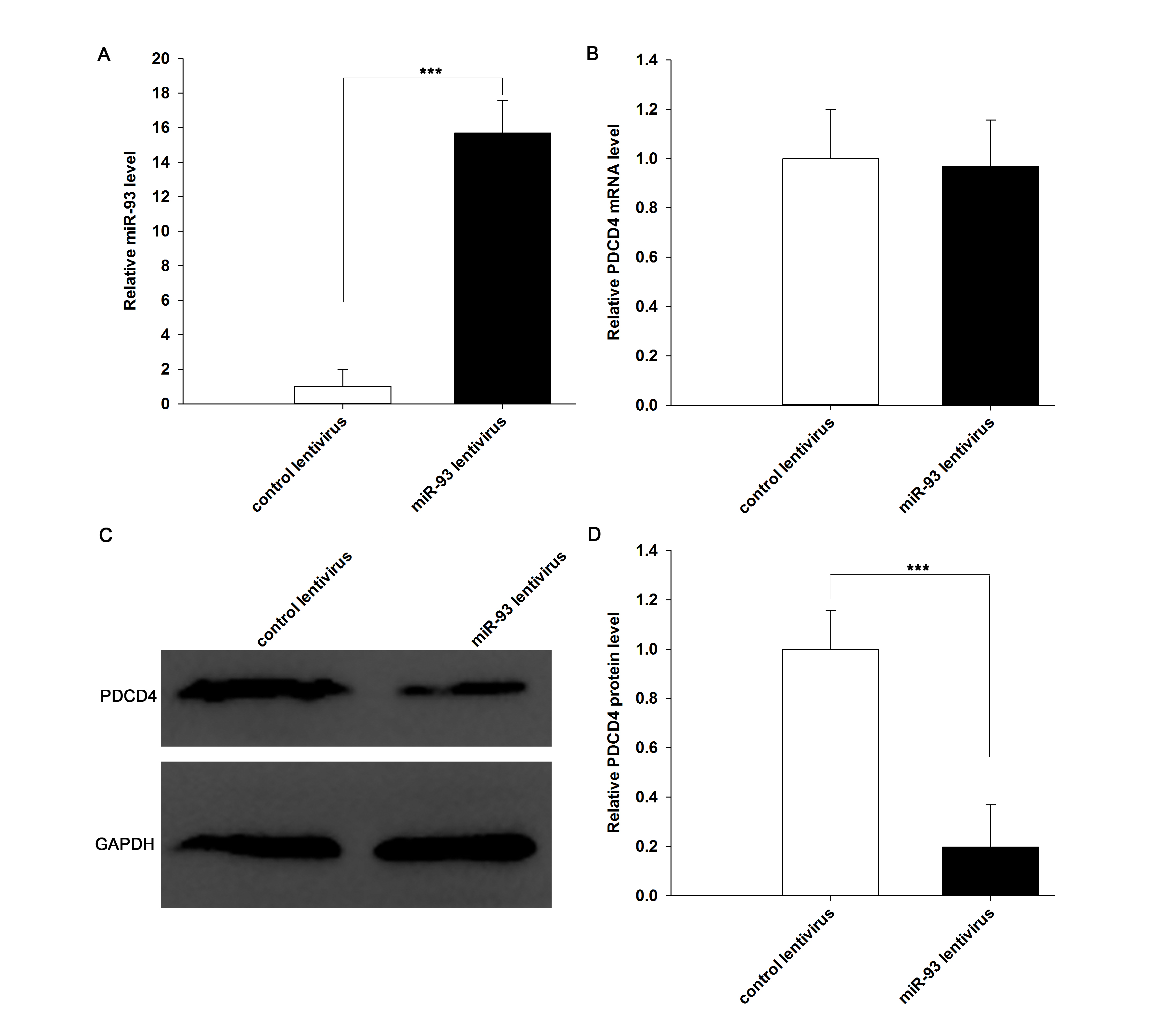

Supplement: Supplementary Information [file srep23772-s1.doc]
